# Some Early Signs in Tabes Dorsalis

**Published:** 1905-10-14

**Authors:** 


### SOME EARLY SIGNS IN TABES DORSALIS.

In a clinical lecture on three cases of tabes dorsalis, Dr. C. O. Hawthorne[1] draws attention to some of the modes in which this disease may first display itself. He emphasises more particularly the proposition that for long periods the patient may suffer inconvenience from only a single symptom, and that this may be one by no means strongly suggestive of central nervous disease. Hence he argues that an ordinary clinical examination ought to include a systematic investigation of the nervous system parallel to what is usually adopted in the case of the thoracic and abdominal viscera. Were this a routine practice many cases having the early beginnings of nervous disease, which are now overlooked or misinterpreted, would be recognised, and a correct and early diagnosis would be found to be possible. For whilst it may often be the case that the patient's subjective experience includes only a single ground of complaint, there may at the same time be objective signs of disease, readily appreciated by the physician though causing no inconvenience to the patient. These positions are illustrated by the facts quoted from various case records. In the first patient the complaint was of attacks of pain in the trunk, limbs, and head, the general health in every other respect being quite satisfactory. These conditions had existed for several years, and such diagnoses as gout, rheumatism, and neuralgia had been suggested. Examination, however, revealed the features of the Argyll-Robertson pupil and the entire absence of the knee and heel-jerks. Thus the case was recognised as one of "tabetic neuralgia," and treatment by aluminium chloride, by hot douches and mustard packs to the spine, and by the administration of acetanilide at the onset of the pain, had been found to give great relief. In a second case the only symptom volunteered by the patient was defective sight in the left eye. This was found to be due to atrophy of the optic nerve. In the right eye the ophthalmoscopic appearances were normal and the visual acuity was 6/6. Nevertheless the perimeter showed a sector-shaped defect in the upper and outer part of the visual field, and this, in all the circumstances, was held to signify that optic atrophy was also commencing in the right eye. The case thus became one of bilateral optic atrophy—a condition in itself suggesting a diagnosis of tabes dorsalis. Further examination revealed Argyll-Robertson pupils, absence of the tendon jerks, and a zone of anæsthesia round the trunk. Thus what was regarded by the patient merely as a defect of vision in one eye was shown to be a quite undoubted case of tabes dorsalis. In the third patient the one ground of complaint was diplopia. This was seen to be due to an extensive paralysis in the area of distribution of the left third nerve. In all other respects the patient regarded himself as in good health, and physical examination yielded entirely negative results. But it was stated that some twelve months ago the patient had suffered for several weeks from a previous attack of diplopia due to paralysis of the sixth nerve. Further, he was forty-six years of age and had suffered without any question from constitutional syphilis. In view of all these facts it was urged that the case could not be regarded disease not infrequently produces ocular paralyses On the contrary, a diagnosis of tabes dorsalis was definitely claimed, and in support of it was quoted the clinical experience which shows that that disease not infrequently produces ocular paralyses which are often of temporary duration and which tend to recur. Further, it was pointed out that if such a paralysis were a first event in the evolution of the disease in an individual patient no symptom was more likely to come promptly under medical observation. Loss of knee-jerks, Argyll-Robertson pupils, optic atrophy (when confined to the eye) may long escape the patient's attention, but double vision at once causes alarm. Hence an ocular paralysis is of all the beginnings of tabes dorsalis the one most likely to receive prompt recognition by the patient, and therefore the most likely to be found without confirmatory evidences in other parts of the body. In view of this, it was claimed that an ocular paralysis in a man of middle age, who had suffered from syphilis and with a recent history of a previous temporary diplopia, must, in spite of the absence of all other evidence, be regarded as the beginning of those degenerative changes which form the pathological basis for the disease known clinically as tabes dorsalis.

[1] Lancet, September 30, 1905.

